# Secretion of extracellular hsp90α via exosomes increases cancer cell motility: a role for plasminogen activation

**DOI:** 10.1186/1471-2407-10-294

**Published:** 2010-06-16

**Authors:** Jessica McCready, Jessica D Sims, Doug Chan, Daniel G Jay

**Affiliations:** 1 Department of Physiology, Tufts University, 136 Harrison Ave, Boston, MA 02111, USA; 2 Protech Laboratory Inc, Houston, TX 77054, USA

## Abstract

**Background:**

Metastasis is a multi-step process that is responsible for the majority of deaths in cancer patients. Current treatments are not effective in targeting metastasis. The molecular chaperone hsp90α is secreted from invasive cancer cells and activates MMP-2 to enhance invasiveness, required for the first step in metastasis.

**Methods:**

We analyzed the morphology and motility of invasive cancer cells that were treated with exogenous exosomes in the presence or absence of hsp90α. We performed mass spectrometry and immunoprecipitation to identify plasminogen as a potential client protein of extracellular hsp90α. Plasmin activation assays and migration assays were performed to test if plasminogen is activated by extracellular hsp90α and has a role in migration.

**Results:**

We found that hsp90α is secreted in exosomes in invasive cancer cells and it contributes to their invasive nature. We identified a novel interaction between hsp90α and tissue plasminogen activator that together with annexin II, also found in exosomes, activates plasmin. Extracellular hsp90α promotes plasmin activation as well as increases plasmin dependent cell motility.

**Conclusions:**

Our data indicate that hsp90α is released by invasive cancer cells via exosomes and implicates hsp90α in activating plasmin, a second protease that acts in cancer cell invasion.

## Background

Approximately 90% of cancer deaths are not from the primary tumor but due to metastasis to distant sites [1]. Current treatments do not target metastatic disease. Towards developing anti-metastasis drugs, a functional proteomic screen was performed to identify surface proteins required for tumor cell invasion, the first step in metastasis [2]. One of the proteins identified was the molecular chaperone heat shock protein 90α (hsp90α) [2]. Intracellular hsp90α aids in the folding, assembly-disassembly and activation of a variety of client proteins including kinases, steroid hormone receptors and transcription factors [3]. We discovered that extracellular hsp90α acts in tumor cell invasion through its activation of the pro-invasive protein matrix metalloproteinase-2 (MMP-2). Since the publication of this study, additional reports in the literature have demonstrated the importance of extracellular hsp90α in both physiological and pathological states. Extracellular hsp90α is required for both dermal fibroblast [4] and neuronal motility [5] as well as for melanoma migration [6], invasion and metastasis [7].

The secretion method of extracellular hsp90α from invasive cancer cells has not been fully elucidated. Hsp90α has been found in exosomes in immune and other physiologically normal cell types [8-11] and suggested to be in exosomes in diabetic cells [12]. Exosomes are small vesicles, approximately 30-100 nM in diameter, that are part of the endocytic pathway. They are secreted as intact vesicles that form within multivesicular bodies (MVB) and are released from cells when the membrane of the MVB fuses with the plasma membrane. Exosomes function in the immune system and in acellular communication [13]. Recent reports indicate that exosomes contribute to the aggressive nature of gliomas by transferring the mutated EGFRvIII receptor between cells [14]. The presence of hsp90α in exosomes of other cells types and the observation that exosomes contribute to glioma aggressiveness suggested to us that hsp90α in exosomes might contribute to cancer invasiveness.

In this study, we demonstrate that hsp90α is secreted from invasive cancer cells via exosomes and increases cancer cell migration. We show that extracellular hsp90α is necessary for the activation of a second extracellular protease, plasmin, and that fibrosarcoma cell movement is dependent on this activation.

## Methods

### Cell culture

A172, HT-1080, and MDA-MB231 cells were obtained from ATCC and maintained in DMEM supplemented with 10% FBS, 1% NEAA, and 1% P/S. SUM159 cells were a kind gift from Charlotte Kuperwasser and were maintained in Hams F12 media supplemented with 5% FBS, 5 μg/mL insulin, 10 ng/mL EGF and 1% P/S. All cells were grown in a 37°C incubator with 7.5% CO_2_.

### Quantitative Real time PCR

Total RNA was extracted from MDA-MB231 breast cancer cell lines with TRIzol (Invitrogen, California) and 2 μg of RNA was reverse transcribed into cDNA with Superscript III (Invitrogen) following the instructions supplied by the supplier. Real time PCR was performed at the Tufts Univesity Center for Neuroscience Research using the Stratagene real time cycler. Primer sequences were as follows: HSP90AA1-1 forward 5'-GGCAGAGGCTGATAAG-AACG-3' and reverse 5'CCCAGACCAAGTTTGATCATCC-3'; HSP90AA1-2 forward 5'-CATCTGATGGTGTCTGGATCC-3' and reverse 5'-AATGGCTGCAGATCCTTGTAG-3'. Samples were analyzed using the 2-ΔΔCT method (29) with GAPDH as the reference.

### Brefeldin A Treatment

MDA-MB231 cells were treated with 10 μg/mL Brefeldin A (BFA), (Sigma, Missouri) or vehicle control for 16 hours. Conditioned media was collected, concentrated and subjected to SDS-PAGE followed by a Western blot probed with MMP-2 antibody (EMD Biosciences, New Jersey), anti-hsp90α or β-actin antibody (Sigma, Missouri). β-actin protein should be absent in conditioned media samples isolated from intact, alive cells.

### RNAi Treatment

MDA-MB231 cells were transfected with either control siRNA (non-targeting) or 100 nM siRNA directed against the HSP90AA1-2 (sense 5'-GTTAACTGGTACCAAGAAA-dTdT-3') isoform using Oligofectamine (Invitrogen). RNA was extracted as indicated above and the results are graphed as percentage knockdown setting the control at 100%.

### Exosome isolation

Exosomes were isolated from A172, HT-1080, MDA-MB231, and SUM159 cells as previously described [8]. Briefly, 5 × 10^6 ^cells were plated in 10% DMEM and allowed to settle overnight. Cells were then washed with HBSS and re-fed with serum free media or serum free media containing 15 nM dimethyl amiloride (Sigma). Media was collected 48 hours after the addition of serum free DMEM and spun at 300 × g to collect any cellular debris. This media was then filtered with a 0.2 μM filter and spun for 1 hour at 110,000 × g. The pellet was washed with PBS and spun for 1 hour at 110,000 × g. One μg of protein was subjected to Western Blot probed for hsp90α (Assay Designs, Michigan). Samples were also probed with an anti-Annexin II antibody (BD Biosciences, California) and Flotillin (Cell Signaling Technology, Massachusetts) as positive controls and vATPase subunit B (Molecular Probes, California) as a negative control.

### Immunostaining

1 × 10^4 ^MDA-MB231 cells were plated into an 8-well chamber slide and treated with exosomes isolated from MDA-MB231 cells or 0.5 μg recombinant hsp90α (Assay Designs) for 16 hours. Cells were fixed in PBS/4% paraformaldehyde/4% sucrose, permeabilized in 0.1% TritonX-100/PBS, blocked in 1%BSA/PBS and stained with Alexa546-labeled phalloidin (Invitrogen, CA) for 30 minutes to visualize F-actin.

### Cell shape analysis

The cell shape and area of MDA-MB231 cells were measured and calculated with OpenLab software (Improvision). Cell shape was defined using the equation (4 × cell area)/cell perimeter^2^, where greater than 1 indicates a perfect circle and values less than 1 indicate a more irregular shape.

### Wound healing assay

1 × 10^5 ^SUM159 breast cancer cells or A172 glioma cells were plated in an 8 well chamber slide. Cells were wounded by scratching a sterile yellow pipette tip lengthwise along the chamber. The cells were washed twice with 1× PBS and serum free media was placed in each well with either the vehicle control PBS, 0.5 μg recombinant hsp90α protein, 1 μg exosomes isolated from SUM159 cells, or 1 μg exosomes isolated from SUM159 cells plus 40 μg/mL anti-hsp90 antibody (SPS-771, Assay Designs). Pictures were taken immediately after cell wounding (0 hours) and 16 hours after cell wounding. Wound width was calculated using OpenLab software and is represented as μm between the cells at 16 hours for each treatment.

### Immunoprecipitation/Mass spectrometry

4 × 10^6 ^MDA-MB231 breast cancer cells were plated in a 150 mm tissue culture dish and allowed to settle for 24 hours. Cells were then refed with serum free media and incubated for 48 hours at 37°C. Conditioned media was concentrated by centrifugation (Millipore, MA) and a protein assay was performed (BioRad, CA). 1 mg of protein was pre-cleared with protein A beads after which 1 ug of hsp90α antibody (Assay Designs) was added to the samples. Samples were washed with RIPA B buffer (50 mM Tris, 150 mM NaCl, 0.5% NP40, 0.25% DOC) boiled, subjected to SDS PAGE, stained with Coomassie Blue and removed from the gel for mass spectrometry analysis. The excised gel bands were analyzed by mass spectrometry as previously described [15]. MS results were verified using antibodies for hsp90α and tPA (Abcam, MA).

### Plasminogen activation assay

Plasminogen activation assays were performed as previously described [16]. Briefly, HT-1080 fibrosarcoma cells were plated in 10% DMEM and refed with serum free media 24 hours after plating. DMSO, 0.5 μM [Glu] plasminogen (American Diagnostica Inc, CT), or 40 μg/mL DMAG-N-oxide (a gift from Len Neckers) were added for five hours at 37°C. DMAG-N-oxide was used in this experiment because the large amount of antibody required for this experiment precluded its use. It has been previously characterized as an inhibitor of extracellular hsp90α [17]. Conditioned media was concentrated (Millipore) and a protein assay was performed (Bio-Rad). 25 μg of each sample was loaded into a 0.1% gelatin zymogram. The zymogram was washed twice for two hours each in wash buffer (50 mM Tris-HCl, 150 mM NaCl, 2.5% (v/v) Triton X-100, pH 7.4), three times for 5 minutes each in water and then incubated in wash buffer for 12 hours at 37°C. The zymogram was stained with 0.5% coomassie, destained, and densitometry was performed to determine the plasminogen activation levels of each condition.

### Migration assay

HT-1080 fibrosarcoma cells were plated in 10% DMEM. 48 hours after plating the cells were labeled with CMTMR (Invitrogen) and 1 × 10^5 ^labeled cells were plated into a 24-well Fluoroblok plate (BD Biosciences, CA). Cells were treated with either 40 μg/mL rabbit IgG, 40 μg/mL anti-hsp90α (SPS-771, Assay Designs), or 0.5 μg plasmin (Molecular Innovations, MI). Cells were allowed to migrate for 24 hours after which the number of cells that migrated to the bottom chamber were photographed and counted.

## Results

### Hsp90α is secreted via exosomes in invasive cancer cells

**Figure 1:**
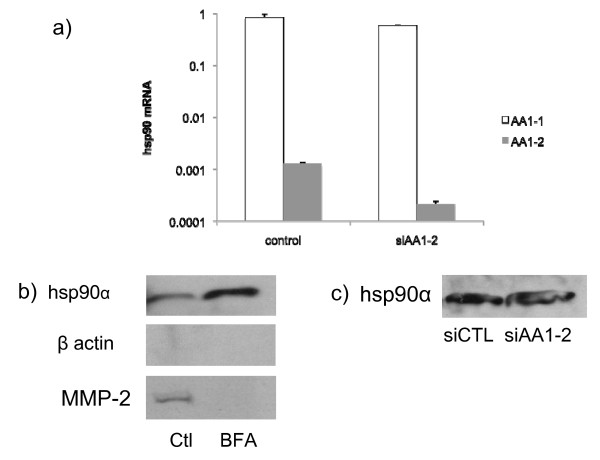
**Hsp90α is not secreted either through the classic secretory pathway or in an isoform specific fashion. **(a) We identified two isoforms of hsp90α in breast cancer cell lines using quantitative real time PCR. Cells were transfected with either control siRNA (non-targeting) or 100 nM siRNA directed against the HSP90AA1-2 isoform. Data from one of the three cell lines tested are represented (A172 and HT1080 not shown). (b) MDA-MB231 cells were treated with BFA or vehicle control for 16 hours and conditioned media was collected, concentrated and subjected to SDS-PAGE followed by a Western blot probed with anti-hsp90α, MMP-2 or β-actin antibody. (c) Conditioned media from cells treated with siRNA directed against the HSP90AA1-2 isoform was collected, concentrated and subjected to SDS-PAGE followed by a Western blot probed with an anti-hsp90α antibody.

While the importance of extracellular hsp90α for tumor cell migration, invasion and metastasis has been recognized, the mechanism that invasive tumor cells use to secrete hsp90α is still unclear. In response to our work (2), it was hypothesized that alternative splicing of hsp90α created multiple isoforms and that perhaps there was a mainly intracellular hsp90α isoform and a mainly extracellular isoform of hsp90α (18). This was of interest because one could then target drugs to the extracellular isoform without interfering with the important intracellular functions of hsp90α. We verified that the two hypothesized Hsp90α isoforms existed in MDA-231 cells and determined the relative amounts using Real Time PCR (Figure 1a). We found that the primary isoform present in MDA-231 cells was the classical ten-exon isoform (AA1-1). The other isoform, AA1-2, with two additional exons, was present in only very small quantities. Even though there was only a small amount of the second isoform present, we hypothesized that this isoform could be targeted outside of the cell since the amount of Hsp90α inside of the cell is much greater than the amount outside of the cell. In order to test if Hsp90α was indeed being exported via a signal sequence in the two extra exons, we treated MDA-231 cells with Brefeldin A, a compound that inhibits the export of proteins through the canonical pathway. We collected the conditioned media from the cells, subjected it to SDS-PAGE and blotted for Hsp90α (Figure 1b). Hsp90α was detected outside of the cell after BFA treatment in both cell lines indicating that it is not exported through the canonical signal sequence pathway. In fact, the extracellular hsp90α protein levels were markedly increased by Brefeldin A, probably in response to cellular stress caused by this inhibitor. As a positive control we probed for MMP-2, a protein known to be exported via a signal sequence, and we found that its secretion was markedly inhibited by BFA (Figure 1b).

**Figure 2:**
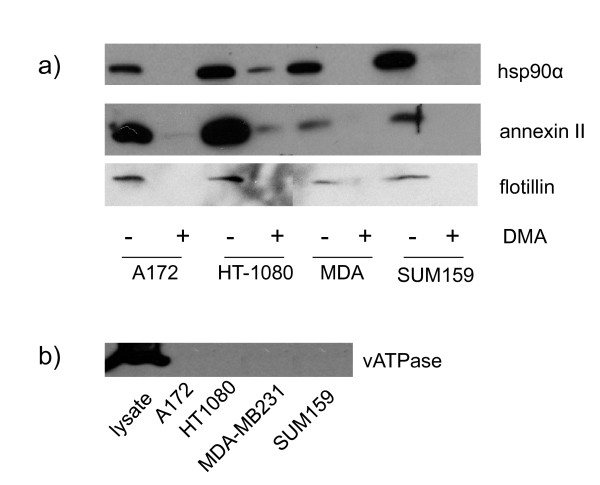
**Hsp90α is present in the exosomes of four cancer cell lines. **Western blot analysis of exosomes isolated from invasive cancer cell lines treated with vehicle (-) or DMA (+), which blocks the exosome pathway. Proteins were resolved by electrophoresis on 10% SDS-PAGE and transferred to a nitrocellulose membrane. Western blot was performed using hsp90α, annexin II, flotillin, and vATPase antibodies. Representative images taken from three independent experiments are shown.

Even though Hsp90α is not exported via a signal sequence, it may still be exported in an isoform specific manner. In order to test this we used siRNA to knock down the AA1-2 isoform. We obtained approximately 80% knock down of this isoform in the cells (Figure 1a) but did not see any reduction in the amount of Hsp90α outside of the cell (Figure 1c). These results indicate that hsp90α is not secreted by the classical secretory pathway or in an isoform specific manner. This led us to explore other non-classical secretory pathways such as exosomes. We isolated exosomes from MDA-MB231 cells and showed by immunoblot that they contain hsp90α (Figure 2a). To test the generality of this, we isolated exosomes from three other invasive tumor cell lines from different lineages, A172 glioblastoma cells, HT-1080 fibrosarcoma cells, and SUM159 breast cancer cells. The exosomes isolated from these cells also contain hsp90α (Figure 2a). Purity of our exosome preparations was verified by the presence of annexin II and flotillin, two markers for exosomes, as well as by the absence of the large B subunit of the v-ATPase (Figure 2b), which is found in lysosomes and plasma membrane but not in exosomes [18]. When cells were treated with dimethyl amiloride (DMA), which blocks the exosome pathway[19], isolated exosome preparations from all cell lines tested showed marked reduction in hsp90α as well as exosome markers (Figure 2a). Both hsp90α and annexin II were degraded in a protease protection assay suggesting that they are easily accessible to extracellular proteins (data not shown). Therefore, hsp90α in exosomes is accessible to activate secreted extracellular proteins such as MMP-2 [2].

#### Exosomes induce a change in morphology of breast cancer cells

**Figure 3:**
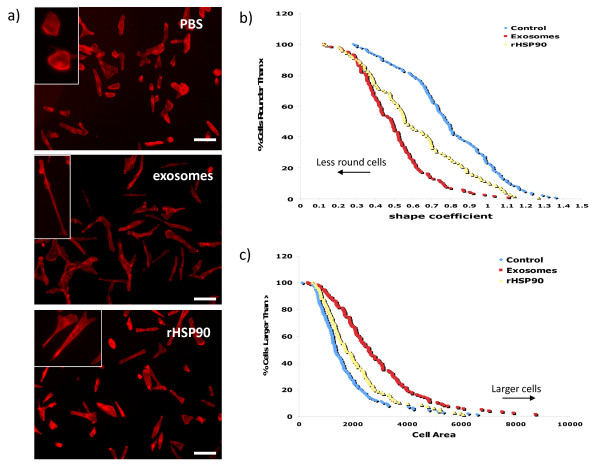
** Exosomes alter the morphology of invasive breast cancer cells. **Immunofluorescence and cell morphological analysis of MDA-MB231 cells. (a) Cells were treated with PBS (top panel), exosomes isolated from MDA-MB231 cells (middle panel) or recombinant hsp90α protein (bottom panel) and stained with phalloidin to visualize F actin. Images taken at 10× magnification from three independent experiments. Scale bar = 100 μM. Inset: Representative images at 20× magnification. (b) Chang plot showing cell shape measurements. Cell shape is defined using the equation (4 × cell area)/cell perimeter^2^, where greater than 1 indicates a perfect circle and smaller values indicate a more irregular shape. Cell shape was calculated using OpenLab software. n = 130. (c) Cell area analysis of MDA-MB231 cells treated with exogenous exosomes or recombinant hsp90α. Cell area was calculated using OpenLab software. n = 130.

Recent reports indicate that cancer cells secrete exosomes containing annexins and major histocompatibility complex proteins, which are normally associated with exosomes but also contain proteins involved in cell adhesion and motility such as integrins and fibronectin [20-22]. These reports, along with our previously published data regarding the role of extracellular hsp90α and tumor cell motility, suggest that exosomes containing hsp90α released by invasive cancer cells could increase tumor cell motility. To test this hypothesis we isolated exosomes from the breast cancer cell line MDA-MB231, added them to the media of MDA-MB231 cells plated the previous day and stained the cells with phalloidin to analyze the morphology of the cells in the presence of exosomes (Figure 3a). Cells exposed to the control vehicle, PBS, display normal morphology, whereas cells exposed to exosomes for 16 hours show a more polarized shape associated with a motile phenotype. We also exposed MDA-MB231 cells to recombinant hsp90α protein to determine if the effect we see with the exogenous exosomes is due in part to the presence of hsp90α in the exosomes. Hsp90α treated cells are more polarized than control-treated cells but less polarized than the cells treated with exosomes. To quantitate these changes, we measured both the cell shape and cell area using the OpenLab software program (Figure 3b). A motile cell can have a more linear cell shape as well as a large cellular area. We measured the perimeter of cells treated with either PBS, recombinant hsp90α protein or exosomes. The data are represented in a Chang plot that depicts the shape of cells in which a perfect circle is greater than 1 and a perfect line is 0. Cells treated with PBS have the most circular shape (average = 0.79 ± 0.02), cells treated with exosomes have the least circular shape (average = 0.49 ± 0.02) and cells treated with recombinant hsp90α show intermediate values between those seen for control and exosome treated cells (average = 0.61 ± 0.03). Cells treated with either the recombinant hsp90α or exosomes are statistically significantly different in shape when compared to the control treated cells (p < 0.001). Exosome treatment also significantly increases the cell area when compared to cells treated with PBS or recombinant hsp90α (Figure 3c). Cell area increased from an average of 1137 ± 121 μm^2 ^to an average of 1398 ± 138 μm^2 ^with the addition of recombinant hsp90α (p < 0.02) and further increased to an average of 1914 ± 167 μm^2 ^with the addition of exosomes (p < 0.001). Taken together these results indicate that exogenous exosomes cause changes in both cell shape and cell area, consistent with motile behavior. We performed this experiment with A172 glioma cells (data not shown), however since they display a polarized morphology under normal growing conditions, the addition of exosomes did not affect their morphology.

#### Exosomes increase cancer cell motility

**Figure 4:**
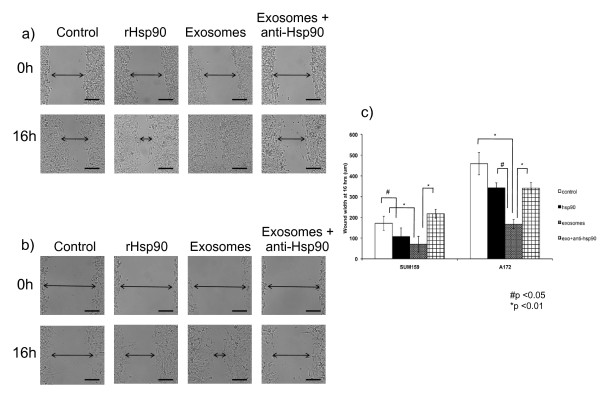
** Exosomes increase hsp90α dependent glioma and breast cancer cell movement. **(a) SUM159 breast cancer cells were wounded and images captured immediately and 16 hours after wounding. Cells were treated with either PBS, recombinant hsp90α protein, exosomes isolated from SUM159 cells or exosomes isolated from SUM159 cells plus anti-hsp90α antibody. Representative images from three independent experiments are shown for each time point and condition. Scale bar = 150 μM (b) A172 glioma cells were wounded and images captured immediately or 16 hours after wounding. Cells were treated with either PBS, recombinant hsp90α protein, exosomes isolated from A172 cells or exosomes isolated from A172 cells plus anti-hsp90α antibody. Representative images from three independent experiments are shown for each time point and condition. Scale bar = 150 μM (c) Graphical representation of the wound healing assay with SUM159 and A172 cancer cell lines. The addition of both recombinant hsp90α protein and anti-hsp90α antibody results in a significant decrease in wound closure at 16 hours (p < 0.05, p < 0.01 respectively, two tailed t test).

To determine if the changes in cell morphology translate to an increase in cell motility we performed wound healing assays. Since MDA-MB231 cells are not well suited to the wound healing assay we used two highly motile and invasive cell lines originating from different tumors: SUM159 breast cancer cells (Figure 4a) and A172 glioma cells (Figure 4b). We imaged the wound immediately after wounding the cells (0 hours) and added either recombinant hsp90α, exosomes, or exosomes plus a function inhibiting hsp90α antibody [4]. We captured a second set of images 16 hours later. SUM159 control cells moved significantly less than cells treated with either 0.5 μg recombinant hsp90α (p < 0.05) or 1 μg exosomes (p < 0.01). The effect of exosomes on SUM159 cells was reduced by the hsp90α function inhibiting antibody suggesting that hsp90α is required for an exosome-dependent increase in cell movement. Control cells moved significantly less than cells treated with either recombinant hsp90α (p < 0.05) or exosomes (p < 0.01). A172 cells acted similarly to SUM159 cells in the wound healing assay. Data from the wound healing assay is represented graphically in Figure 4c.

#### Extracellular hsp90α immunoprecipitates with tissue plasminogen activator

**Figure 5:**
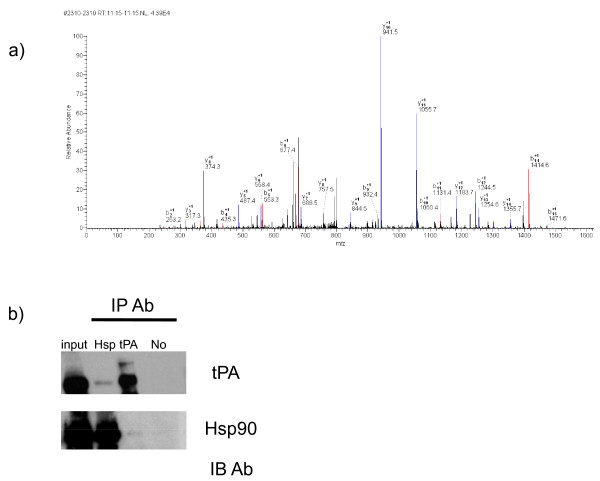
**Extracellular hsp90α co-immunoprecipitates with tissue plasminogen activator. **(a) MS/MS spectrum of the peptide (^248^VYTAQNPSAQALGLGK^263^) that was used to identify tPA as a HSP90α interacting protein. (b) Immunoprecipitation of hsp90α from the conditioned media of HT-1080 fibrosarcoma cells. Hsp90α was immunoprecipitated from the media of HT-1080 cells and resolved on 10% SDS-PAGE. Western blot analysis was performed using anti-hsp90α or anti-tPA antibodies. The input lane contains approximately 1/100^th ^of the total media collected. "IP Ab" indicates the antibody used during immunoprecipitation (hsp90α, tPA or no antibody as the negative control). "IB Ab" indicates which antibody was used the probe the immunoblot. Representative images from three independent experiments are shown.

Our findings established that the addition of exogenous exosomes changed both the morphology and motility of cancer cells. We had previously implicated hsp90α in the activation of MMP-2, but it is possible that hsp90α secreted via exosomes could activate other extracellular proteins. We hypothesized that extracellular hsp90α was interacting with other extracellular proteins to increase cancer cell motility. To identify proteins that associate with hsp90α we performed immunoprecipitation with conditioned media from MDA-MB231 cells followed by mass spectrometry (Figure 5a). We discovered ten proteins bound to extracellular hsp90α. The identified proteins are in their precursor form suggesting that they might be potential clients for this chaperone. We focused on one that could be linked to both exosomes and cell motility: tissue plasminogen activator protein (tPA). Annexin II, a protein secreted via exosomes, binds to both tPA and plasminogen and has been shown to associate with hsp90α [23]. This binding initiates conversion of plasminogen to the protease plasmin [24]. We verified the mass spectrometry result with co-immunoprecipitation of Hsp90α and tPA in conditioned media from HT-1080 fibrosarcoma cells (Figure 5b), the cell line for which we originally discovered the interaction of extracellular hsp90α and MMP-2, and MDA-MB231 cells (data not shown). tPA and hsp90α weakly interact in both MDA-MB231 and HT-1080 cells perhaps because client and chaperone proteins interact transiently [25]. This raises the possibility that tPA is a novel client protein for extracellular hsp90α.

#### Hsp90α aids in the conversion of plasminogen to plasmin

**Figure 6:**
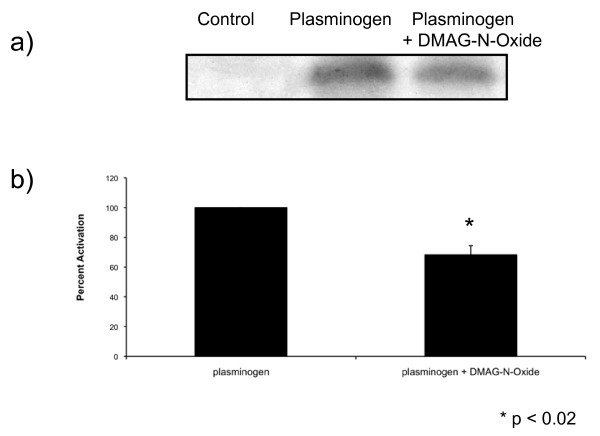
** Extracellular hsp90α assists in the conversion of plasminogen to plasmin. **(a) HT-1080 fibrosarcoma cells were treated with either DMSO, plasminogen or plasinogen plus DMAG-N-oxide, a membrane impermeant inhibitor of hsp90α. Samples were added to a gelatin zymogram. A representative image taken from three independent experiments is shown. (b) Graphical representation of the plasminogen activation assay. Densitometry results are expressed as the percent decrease in plasmin activation (control set to 100%) and are from three independent experiments. Inhibition of extracellular hsp90α with DMAG-N-oxide results in a 32% decrease in plasmin activation (p < 0.02, two tailed t test).

Previous data from our lab indicated that extracellular hsp90α increases cancer cell invasion by assisting in the activation of MMP-2 [2]. To test if extracellular hsp90α can promote the activation of other extracellular proteins involved in cancer cell motility we assessed whether the activation of plasmin through its association with tPA requires extracellular hsp90α. We performed plasmin activation assays with HT-1080 fibrosarcoma cells in the presence or absence of an inhibitor of extracellular hsp90α (Figure 6a). We used DMAG-N-oxide, an impermeable form of the hsp90α inhibitor geldanamycin [17] to determine if extracellular hsp90α activates plasmin. The addition of DMAG-N-oxide resulted in a 32% decrease in activated plasmin when compared to cells that were treated with vehicle alone (Figure 6b, p < 0.02). These findings indicate that extracellular hsp90α is involved in the conversion of plasminogen to plasmin.

#### Inhibition of extracellular hsp90α decreases tumor cell migration

**Figure 7:**
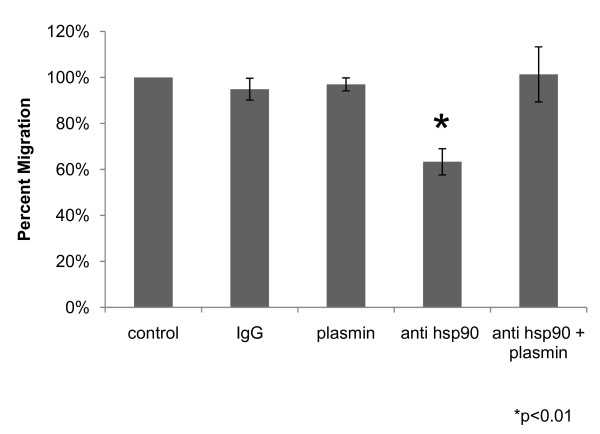
**Inhibition of extracellular hsp90α inhibits cancer cell motility by preventing plasmin activation. **HT-1080 cells were treated with control, IgG, plasmin, anti-hsp90α antibody, or plasmin plus anti-hsp90α antibody. A transwell migration assay was performed and cells that migrated through the chamber were counted. Results are from four independent experiments and expressed as percentage decrease from control (100%). Anti-hsp90α antibody results in a 37% decrease in migration (p < 0.01, two tailed t test).

While extracellular hsp90α can activate plasmin, it was not known if this activation contributes to increased tumor cell motility. We performed transwell migration assays using a function-inhibiting antibody against hsp90α (Figure 7). This antibody will only inhibit the extracellular hsp90α because antibodies are membrane impermeant. HT-1080 fibrosarcoma cells treated with the hsp90α antibody migrated 37% less than control treated cells (p < 0.01). Interestingly, the addition of plasmin alone did not increase cell motility. Perhaps the cells are already saturated with active plasmin such that additional plasmin would not affect migration. Normal migration was recovered when we added activated plasmin to the cells treated with hsp90α antibody. Together, these findings indicate that hsp90α can activate plasmin and this activity stimulates cell motility.

## Discussion

In this study we present evidence that extracellular hsp90α, secreted via exosomes, activates a novel client protein and increases tumor cell motility. Previously published work from our lab and others indicate that extracellular hsp90α contributes to the activation of both MMP-2 [2] and HER-2 [26], two proteins involved in cancer metastasis. We now present data indicating that extracellular hsp90α is necessary for the activation of a second protease, plasmin, also involved in tumor metastasis [27]. Inhibiting extracellular hsp90α *in vivo *inhibits both wound healing [4] and tumor invasion [17]. Our current findings suggest however that other exosomal proteins may also contribute to these processes. Hsp90α protein alone does not elicit as complete an effect on cell morphology or movement as the addition of exosomes (Figures 3 and 4). Hsp90α binds to tPA and inhibiting hsp90α decreased plasmin activation and cell migration. Therefore, we speculate that hsp90α is part of an extracellular complex including annexin II, tPA and plasminogen that functions to increase cell movement. Annexin II is found in exosomes and has an established role in aggressive tumors and binds both tPA and plasminogen thereby enhancing the conversion of plasminogen to active plasmin [24]. Extracellular hsp90α increases annexin II at the cell surface in rat aortic cells, leading to an increase in plasmin production in these cells [23]. Also, cell surface annexin II expression levels are increased in metastatic tumors and it interacts with multiple extracellular proteases that have been implicated in tumor progression [28]. Although plasmin is known for its role in cellular invasion it has not been well studied in migration, one component of the multi-step process of tumor invasion. Plasmin may be contributing to cell migration by contributing to the local remodelling of the extracellular matrix exposing cryptic cell attachment sites necessary for cellular migration, similar to that seen in smooth muscle cells during wound healing [29]. It is also possible that plasmin contributes to cell migration by interacting with currently unknown targets.

We suggest that exosome contents are released outside the tumor cell in close proximity to each other and other inactive extracellular pro-invasive proteins such as plasminogen. Once released from the exosomes, extracellular hsp90α assists in the activation of pro-MMP2 as well as plasminogen. Beyond MMP-2 and plasmin it is possible that hsp90α could activate other extracellular proteins as most of the proteins identified by mass spectrometry in this study were found in their inactive pro-forms. It is therefore interesting to speculate that extracellular hsp90α could activate a cassette of proteins that function collectively in cancer cell migration. These proteins would act in concert to enhance breakdown and remodeling of the extracellular matrix and permit the tumor cell to invade its microenvironment. Thus, inhibition of extracellular hsp90α could inhibit a growing number of proteins that are responsible for increased tumor cell movement making extracellular hsp90α an attractive target for drug therapy to limit tumor invasion.

## Conclusions

In summary, we have identified that exosomes increase cell motility. One mechanism for this increased motility is the activation of plasmin by extracellular hsp90α. The discovery of a second protease activated by extracellular hsp90α suggests the possibility that the one role of extracellular hsp90α in cancer cells is the activation of precursor proteins that contribute to cellular migration and invasion.

## Competing interests

JM, JDS, DC, DGJ: none declared.

## Authors' contributions

JM contributed to study design, data interpretation, carried out all of the experiments and drafted the manuscript. JDS contributed to experiments in Figure 1, to study design, data interpretation and helped revise the manuscript. DC carried out the mass spectrometry. DGJ contributed to study design, data interpretation and editing of the manuscript. All authors have read and approved the final manuscript.

## Pre-publication history

The pre-publication history for this paper can be accessed here:

http://www.biomedcentral.com/1471-2407/10/294/prepub
